# Community engagement and building trust to resolve ethical challenges during humanitarian crises: experience from the CAGED study

**DOI:** 10.1186/s13031-020-00313-w

**Published:** 2020-10-06

**Authors:** Getnet Yimer, Wondwossen Gebreyes, Arie Havelaar, Jemal Yousuf, Sarah McKune, Abdulmuen Mohammed, Dónal O’Mathúna

**Affiliations:** 1 Global One Health initiative, The Ohio State University, Columbus, OH USA; 2 Department of Animal Sciences, University of Florida, Gainesville, FL USA; 3 Department of Rural Development and Agricultural Extension, College of Agriculture and Environmental Sciences, Haramaya University, Harar, Ethiopia; 4 College of public health and Health Professions, University of Florida, Gainesville, FL USA; 5 College of Nursing, The Ohio State University, Columbus, OH USA

**Keywords:** Ethiopia, Community advisory board, Humanitarian research, Ethics, Trust, Conflict

## Abstract

**Background:**

According to the Internal Displacement Monitoring Centre report on global human displacement, Ethiopia has the highest number of newly displaced people forced to flee their homes. Displaced people have arrived in other regions, sometimes leading to conflict. Several regions in Ethiopia experience on-going ethnic tensions and violence between tribes, which leaves smallholder farmers suspicious of any outside activities in their locale, assuming other ethnic groups may harm them. Changes in the central Ethiopian government have also led to suspicion of non-local agencies. The *Campylobacter* Genomics and Enteric Dysfunction (CAGED) research project’s objective is to improve the incomes, livelihoods and nutrition of smallholder farmers and was conducted during this period of increasing violence. The project aims to assess the impact of reducing exposure to chicken excreta on young children’s gut health and growth. This paper does not report empirical findings from CAGED, but is part of a series that aims to identify challenges in humanitarian research and reports on mitigation strategies during this research.

**Discussion:**

This research is important to determine whether *Campylobacter* infection in chicken’s contributes to illness and stunting in children. However, violence against other researchers in different parts of Ethiopia led to mistrust and lack of engagement by the community with the researchers. Some reactions were so hostile that the team was fearful about returning to some households. As a result, the team designed strategies to respond, including establishing two types of community advisory boards. One used pre-existing village elder structures and another was composed of village youth. Data collection team members received training in principles of ethics, consent, and crisis management, and were provided on-going support from local and international principal investigators and the study’s ethics advisor.

**Conclusion:**

The hostility and mistrust led to fear among the data collectors. These and the resulting strategies to address them resulted in delays for the research. However, the interventions taken resulted in successful completion of the field activities. Moreover, the lessons learned from this project are already being implemented with other projects being conducted in various parts of Ethiopia.

## Background

### Humanitarian context

The *Campylobacter* Genomics and Enteric Dysfunction (CAGED) formative research project was conducted in Haramaya Woreda (or district) in Eastern Ethiopia while conflict occurred in various parts of Ethiopia. According to data from the Internal Displacement Monitoring Centre (IDMC) for the first half of 2019, Ethiopia had the highest number of people newly displaced within their own country due to violence [1]. Conflict has uprooted some 1.4 million Ethiopians from the total population of 107 million. In 2018, the central government changed after being ruled by another party for decades. While the new government is working to bring about peace and security, internal conflict continues partly due to intertribal strife, which makes both routine and humanitarian research very difficult. Although Haramaya was not directly affected by human displacements, the participants’ attitudes and trust levels were impacted by displaced people arriving in nearby regions.

This paper does not report empirical findings from CAGED, but is part of a series that aims to identify challenges in humanitarian research and reports on mitigation strategies during this research. These strategies are supported by a growing body of literature, some of which will be cited. However, an extensive literature review is not within the scope of this paper as it is an individual case study following a specific “Research in Practice” format for this series.

### Research study

The demand for animal source foods (ASF) in Africa is growing rapidly. According to the United Nations, the demand for meat, milk and eggs in Africa will quadruple by 2050, mostly due to population growth and rising living standards [2]. This creates opportunities for improved nutrition and income generation, particularly among Africa’s poorest who produce much of the continent’s ASF.

At the same time, animals also contribute to human illness. One long-term goal of CAGED is to improve the understanding of the causes and mitigation of environmental enteric dysfunction (EED). EED causes stunting in children by severely curtailing the absorption of nutrients in children who ingest fecal droppings from poultry and other livestock in smallholder farms. The CAGED project is led by the University of Florida in collaboration with Haramaya University in East Hararghe zone, Oromia Region, Ethiopia, the Global One Health initiative (GOHi), an affiliate of The Ohio State University with collaborators in Ethiopia, and Washington University in St. Louis. Data collection is led by Haramaya University, with assistance and training provided by the US universities.

Research activities in the CAGED project are set in the context of the Ethiopian government’s Livestock Master Plan (LMP), which aims to increase poultry production significantly by transitioning from traditional (scavenging) family poultry to improved (semi-scavenging) family poultry systems [3]. However, animal excreta, and particularly chicken manure, is considered one of the key factors driving exposure to and (asymptomatic) infection by enteric pathogenic bacteria, and causing EED.

Studies estimate that EED may cause up to 40% of stunting in the developing world [4]. The Etiology, Risk Factors, and Interactions of Enteric Infections and Malnutrition and the Consequences for Child Health (MAL-ED) study identified a high *Campylobacter* prevalence in primarily asymptomatic children in eight low-resource settings, not including Ethiopia. Cumulative *Campylobacter* burden was associated with lower length-for-age Z-scores (LAZ), increased intestinal permeability, and intestinal and systemic inflammation at 24 months of age [5]. The CAGED hypothesis is that *Campylobacter* bacteria are a primary factor in inducing EED through exposure to livestock (predominantly chickens), which are a major reservoir of *Campylobacter*. It is further hypothesized that adequate management of livestock feces will improve childhood nutrition in Ethiopia, particularly within the context of increasing poultry production.

The CAGED project has several phases, some of which are completed and others on-going. Results are currently being written up for dissemination. An ethnographic study was conducted to understand relevant local community contexts, beliefs and practices, and explore the opportunities and challenges with the subsequent research phases. Formative research was conducted around three themes: microbiology, EED and stunting, and agriculture and environment. The formative research aimed to guide decisions about later phases of CAGED by collecting data on colonization of young children and animals with *Campylobacter*. Excreta from chickens and children were collected and tested in Ethiopia and the US for the presence of microbes. Data were collected on nutrition, health, sanitation and social environment of children, as well as animal husbandry practices. The results are informing a longitudinal study to further evaluate risk factors for childhood colonization with *Campylobacter* bacteria, and identify the reservoirs and contamination networks of these bacteria and their impact on young children’s gut health and growth.

In the ethnographic phase of CAGED (March to May 2018) teams from Haramaya University and the University of Florida developed working relationships at the community, regional, and national levels. Data collectors were recruited who were born and raised in the region, knew the local language and culture, and had prior experience working in these localities. The baseline data were collected using participant observation, in-depth, un-structured and semi-structured interviews, and focus group discussions. These methods were used to fully understand a number of pre-identified themes required for appropriate design of the cross-sectional study. This phase gave a detailed understanding of current chicken husbandry practices in the region, and how these may change with LMP implementation.

The formative research (October to December 2018) included a randomised cross-sectional survey to collect information on the prevalence of *Campylobacter* in children approximately 14 months old and livestock in their homesteads, measuring gut health and collecting anthropometric data by measuring linear growth (weight and length) and middle-upper arm circumference. Extensive data were collected through structured household interviews with children’s parents about food consumption, feeding practices, child health, water and sanitation, assets, livestock ownership, and management and women’s empowerment.

The study also involved laboratory analysis of fecal and urine samples that was conducted partly at Haramaya University and partly by US partners as some of the techniques were not available in Ethiopia. All laboratory samples were labelled with codes to anonymize the sources of the samples. This also meant that individual results were not provided to participants, which was explained to families before they agreed to participate. The study team is committed to communicating the aggregated results of the study to the local communities.

#### Ethical aspects

The CAGED study was conducted in accordance with the Declaration of Helsinki ethics standards for clinical trials [6]. Prior to data collection, Institutional Review Board (IRB) approval was obtained from all university partners and the Ethiopian National Ethics Review Committee. Potential participants who were sick were excluded from the study, pertinent findings were communicated to families and they were referred to nearby health care facilities.

### Scientific importance of the research

The overall objective of the CAGED project is to improve the incomes, livelihoods and nutrition of smallholder farmers by increasing the productivity of their livestock through quality feeds and to determine the cause of environmental enteric dysfunction, which causes stunting of poor children, and develop mitigation strategies. Given Ethiopia’s livestock policies and the move to family poultry systems, understanding the health implications of these changes in their humanitarian settings is important. CAGED aims to determine the extent to which *Camplyobacter* contributes to EED and stunting, given that unclean water, poor sanitation and contaminated food are contributing factors also.

### Challenges to the research and resulting research strategies

#### Security and trust

Field data collection was conducted from March to December 2018, with the ethnographic phase from March to May 2018 and the formative phase from October to December 2018. The data collectors visited various kebeles (the Ethiopian term for the smallest administrative units) to collect saliva, urine and feces samples from primary school children and collect household information via surveys, interviews and observation. At the same time, Ethiopia’s central government administration and leadership changed unexpectedly, which impacted the community’s attitude towards any activity involving government offices, including research. Moreover, in October 2018, between enrolment and data collection, an incident happened in another part of the country where three researchers became victims of violence. Problems began after rumors circulated that researchers gave injections and vaccines to children, some of whom got ill and died [7]. A mob dragged the researchers from the school where they were collecting samples and stoned them. Two died and one was critically injured. The incident became widely known and was amplified through social media, and contributed to growing tension and suspiciousness between different ethnic groups.

This incident highlights the ethical significance of trust and its fragility in insecure settings. Trust is especially important when the parties involved have different levels of power, knowledge or influence, as if often the case with humanitarian research [8]. Central to trust is confidence that those being trusted are committed to the best interests of the others, and that they will be faithful to their commitments [9]. Trust thus depends on what people know or believe about the ethical character traits of others, and hence takes time to build as well as a commitment to relationships [10]. Following periods of conflict, trust is often undermined, especially in anyone associated with the authorities. For example, the response to Ebola virus disease in the Democratic Republic of the Congo (DRC) in 2019, as it was in Western Africa in 2014–2016, has been hampered by mistrust. Communities do not trust those arriving in response to Ebola, including researchers, because previous interventions by government officials, the United Nations and humanitarian organizations have failed to bring peace or healthcare services [11].

The CAGED researchers were received well in some villages, but not in others. In some villages, parents who had agreed to enroll in the study decided to discontinue their participation. Some seemed to assume that the researchers were from the government which was not trusted at the time. During the 2013–2016 Ebola outbreak, mistrust between authorities and communities was also common, and engagement with trusted community leaders was found to be crucial [12]. Hence,

CAGED adopted various strategies aimed at building trust. In some villages, the research team was able to meet with the village leadership and spent time together in prayer and other informal activities. The elders learned that the researchers were from the same religion and spoke the same local language. The elders saw that the CAGED research team was from Haramaya University, which is known in the area for its social responsibility-related activities. Also, the local principal investigator (PI) for CAGED is the acting President of the University and is highly respected in Haramaya. The researchers were also able to demonstrate clearly that the team was from the University, not the government. However, in other villages, suspicions were very high, mainly among the youth. The data collectors applied what they had learned in the previous villages, sharing information about themselves and the origins of the research, and prayed with the community. Once trust developed, the research was able to proceed.

As the researchers spent more time in the community getting to know one another and developing trust, community members asked the researchers to take on additional roles in providing services. Researchers are regularly asked to provide relief and aid, which results in ethical dilemmas for the researchers [8]. For example, sometimes severely malnourished children were identified during CAGED data collection. The research team decided to provide medical assistance where appropriate, and transportation to and from nearby health facilities where necessary. While this contributed to better relationships with the community, it also blurred the boundaries between the data collectors’ roles as researchers versus service providers. This can lead to misconceptions over participation, especially if the communities believe they must participate in order to receive the other benefits. In such cases with CAGED, it was decided that the children’s health outweighed these concerns when not providing such help would have serious negative consequences for the children [13]. Research with vulnerable populations should benefit those communities, but finding the correct ethical balance that avoids undue inducement can be challenging [14]. The “dual imperative” of conducting rigorous research and benefiting local communities requires ongoing, transparent engagement between all involved [15].

#### Two types of community advisory boards (CABs)

A more formal strategy developed to assist on-going trust issues. Community advisory boards (CABs) help promote engagement and trust-building between researchers and participant communities, especially in low and middle-income countries. Different approaches are used to form CABs with different member compositions, but two models are generally used to give CABs with either broad representation from across the community or specifically targeted populations [16]. CAGED developed a blended approach in establishing two CABs. One was developed from pre-existing indigenous leadership structures composed mostly of village elders (the Elder CAB). The second was made up of younger members of the community (the Youth CAB).

The data collectors identified greater mistrust between youth and the research team. The youth, through their representative, were invited to form a small group (the Youth CAB) to meet with the research team. The Youth CAB selected a representative to accompany the researchers as they visited each kebele to inform householders about CAGED. This gave researchers time to get to know the representative as they ate, travelled and prayed together. The youth representatives were compensated for their time with the research team.

Using the Youth CAB representative raised ethical challenges. One involved protecting the privacy and confidentiality of the households who were invited to participate in CAGED. This was done by limiting the representative’s role to introducing the researchers to the households and addressing their initial questions. The representative left the house before a decision was made to participate or not. This meant that the representative was not aware of which households participated in the research and which did not. Another challenge arose over expectations regarding the amount of compensation for the representative. Once it was explained by the research team that this payment had not been in the original budget for the study, and that the study had a limited budget which should be focused on bringing good to the community, mutual agreement was reached on the level of compensation. As trust developed, the data collectors were able to conduct their work more easily.

#### Challenges with language

While the above strategies addressed challenges at the community level, other challenges arose at the household level. Seven have refused to participate after initial consenting (which was expected, but the number of refusals after initial consent), and some families threatened the data collectors. Some argued about the wording of the consent form and asked for additional explanations. It came to light that although the consent forms were provided in local languages, one word had a very different meaning in the dialect of a specific locality. Some families requested more time to think about the study, which was uncommon for this kind of study, but completely acceptable to the CAGED team. Other families argued about why stool samples needed to be shipped abroad, which was unusual as samples usually are analyzed more locally. Once the reasons were provided, the language differences resolved, and it was made clearer that participants had the right to withdraw from the study at any time, recruitment improved.

#### Risks to researchers and data collectors

Risk assessments are standard aspects of research ethics analyses, but these have primarily focused on the risks to participants [17]. Some qualitative research has identified a number of risks to which researchers are exposed, especially when addressing sensitive topics [18]. However, as humanitarian work is increasingly informed by research and evidence, more will be conducted in conflict settings. The ethical imperative to do no harm should include harm to researchers [10]. As one experienced conflict researcher observed, “my presence in a war zone was a political act even if I had not intended it that way” ([19], 103). Therefore, the risks to those conducting research during conflict and violence need to be examine carefully and mitigation strategies developed to lower those risks. Campbell [20] provides an extensive review of strategies taken in other projects. This area also needs to include the emotional impact of unrest and violence on researchers, particularly their amplification through social media. Some CAGED researchers reported that the tension and distrust was very difficult emotionally for them, and even if short-lasting they were deeply impacted by these experiences.

A key strategy in working through these dilemmas was training and preparation. The lead field coordinator of the study had prior training in conflict resolution and community engagement, as well as hands-on experience in similar circumstances. He helped train all data collectors on how to manage similar situations, which helped reassure the data collectors about their safety and fostered better engagement with the community. As data collectors moved from one kebele to another, they used each experience as an opportunity to learn and adapt for subsequent sites. The project manager introduced end-of-day review sessions for each team to discuss challenges and lessons learned each day. Moreover, data collectors received training in principles of ethics, consent and conflict resolution. A structured study monitoring tool was used regularly by two GOHi team members to monitor and audit data collection work.

As challenges arose, a system of real-time problem identification was set up where data collectors called their supervisor in Ethiopia, who then contacted the overall CAGED PI at the University of Florida for discussion and advice as needed. This reassured the data collectors that they had easy access to real-time support with the ethical, interpersonal or methodological aspects of the study.

## Conclusions and lessons learned

The negotiations and resulting adjustments, along with the various challenges, took considerable time and resources to mitigate. Given that the study was set up for a limited time period with a fixed budget, members of the research team had to work very hard to adjust to the unexpected developments.

Reflecting on the overall field activity, data collectors considered this experience challenging and difficult. Strategies are required to predict when such problems are likely to occur, and mitigation strategies should be planned from the beginning of a project. When specific challenges arise, these can be adapted to the specific circumstances and settings. Strategies and protocols to develop trust and resolve conflict should be planned, but need to be evaluated to provide evidence regarding which of these are effective.

The ethics training developed for data collectors and the resulting strategies developed in the field helped to build trusting relationships with these communities. These experiences are already being used to inform other studies that Haramaya University is currently conducting. The process of sharing these practices was facilitated by the local PI of the project, as he is also the acting president for research in the University, thus also in charge of advising other research projects on procedural issues and best practices. As political stability in Ethiopia improves, the receptiveness of the community to research is expected to increase.

## Data Availability

The funder of the projet and PLOS have open access policies, so our data will become publicly available if the manuscripts we are currently preparing have been accepted. We have not yet deposited our data.
